# [Fe-EOB-tCDTA] generates strong contrast in the blood but not in the liver, despite inhibiting cellular [Gd-EOB-DTPA]^2-^ uptake and partial liver excretion

**DOI:** 10.1186/s41747-026-00731-0

**Published:** 2026-05-13

**Authors:** Fei Ni, Akvile Haeckel, Hamidreza Hojjat, Mathias Schannor, Heike Traub, Eyk Schellenberger

**Affiliations:** 1 Department of Radiology, Charité–Universitätsmedizin Berlin, Charitéplatz 1, Berlin, Germany; 2 Bundesanstalt für Materialforschung und -prüfung (BAM), Berlin, Germany

**Keywords:** Contrast media, Iron chelating agents, Liver, Magnetic resonance imaging, Renal insufficiency

## Abstract

**Objective:**

Iron-based contrast agents (IBCAs) have potential as alternatives to Gd-based contrast agents (GBCAs), intending to address the long-term safety concerns associated with gadolinium. We investigated [Fe-EOB-*t*CDTA] as a potential alternative to [Gd-EOB-DTPA]^2^^-^ for liver magnetic resonance imaging (MRI).

**Materials and methods:**

[Fe-EOB-*t*CDTA] was synthesized by reacting the monoanhydride of *t*CDTA with 4-ethoxybenzylamine followed by iron chelation. Its kinetic stability was spectrophotometrically evaluated using a zinc stress test. The T1 relaxivity was measured in water and serum at 1.41 T, 37 °C and 3 T, 23 °C. Cellular cytotoxicity against liver-derived BRL-3A cells was evaluated by 3-(4,5-dimethylthiazol-2-yl)-2,5-diphenyltetrazolium bromide assays. The uptake of [Fe-EOB-*t*CDTA] by liver cells was investigated using LA-ICP-MS, in competition with [Gd-EOB-DTPA]^2^^-^. T1 contrast effects in BALB/c mice were evaluated by DCE-MRI.

**Results:**

[Fe-EOB-*t*CDTA] exhibited higher kinetic stability than [Fe-(*t*CDTA)]^-^ and demonstrated a *r*1 of 1.94 and 2.45 mM^-1^s^-1^ at 1.4 and 3 T in serum. No significant differences in the short-term cytotoxicity were observed between [Gd-EOB-DTPA]^2^^-^ and [Fe-EOB-*t*CDTA]. [Fe-EOB-*t*CDTA] inhibited [Gd-EOB-DTPA]^2^^-^ uptake in BRL-3A liver cells. [Fe-EOB-*t*CDTA] (0.2 mmol/kg) demonstrated a comparable blood peak RE% compared to [Gd-DO3A-butrol] (0.1 mmol/kg). However, RE of [Gd-EOB-DTPA]^2-^ in liver at a clinical dose was significantly higher than that of [Fe-EOB-*t*CDTA] at both injection doses.

**Conclusion:**

[Fe-EOB-*t*CDTA] provides comparable blood enhancement to [Gd-DO3A-butrol] and exhibits hepatobiliary excretion like [Gd-EOB-DTPA]^2^^-^ but without a comparable liver contrast. [Fe-EOB-*t*CDTA] may serve as an alternative to nonspecific GBCAs, particularly for patients with renal insufficiency and a contraindication to GBCAs.

**Relevance statement:**

This study reports on the synthesis and *in vitro* and *in vivo* characterization of the iron complex [Fe-EOB-*t*CDTA]. This complex demonstrates strong blood contrast and liver excretion, though it lacks strong liver contrast. This complex could serve as an extracellular contrast agent, particularly for patients with reduced kidney clearance.

**Key Points:**

- [Fe-EOB-*t*CDTA] was synthesized as a novel iron-based MRI T1 contrast agent with liver excretion.
- [Fe-EOB-*t*CDTA] generated a strong blood enhancement effect and exhibited hepatobiliary excretion.
- Negative charge is crucial to the hepatic uptake and long-lasting liver enhancement.

**Graphical Abstract:**

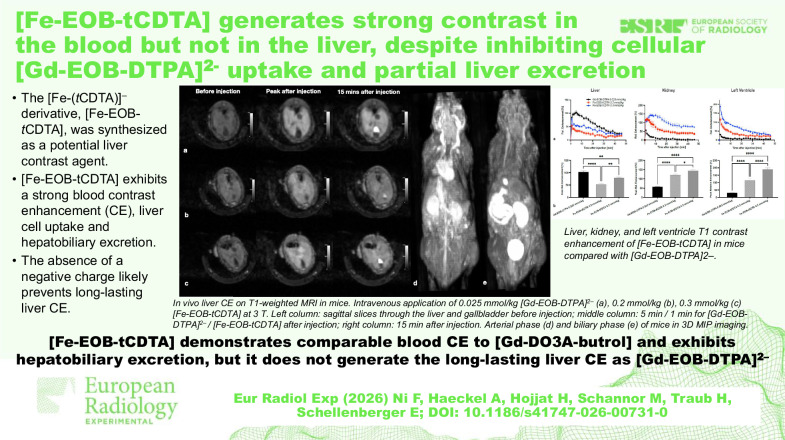

## Background

Magnetic resonance imaging (MRI) is widely used in clinical diagnostics, providing high-resolution soft tissue contrast and noninvasive visualization of anatomical and pathological structures. Gadolinium-based contrast agents (GBCAs) play a key role in enhancing the diagnostic capabilities of MRI and are used in approximately 40% of patient scans tens of millions of times annually in clinical settings [1]. However, since the identification of nephrogenic systemic fibrosis as a rare but severe side effect of less stable GBCA in patients with renal impairment, gadolinium deposition in the brain, skin, bone, and other tissues, even for stable macrocyclic GBCA in patients with normal renal function, has raised concerns about the potential long-term toxicity [2–6]. In particular, linear agents pose a substantially higher nephrogenic systemic fibrosis risk than macrocyclic agents and exhibit greater tissue deposition [7–11]. Due to similar atomic size, gadolinium can block calcium binding sites and may interfere with processes regulated by calcium, *e.g*., affecting patients’ immune responses [12]. Consequently, these concerns have prompted the regulatory agencies to revise their recommendations for the use of GBCA and recommend only the use of the more stable macrocyclic GBCAs for intravenous application, with two exceptions: The less stable linear GBCAs gadoxetic acid ([Gd-EOB-DTPA]^2^^-^, Bayer HealthCare Pharmaceuticals) and gadobenate dimeglumine (Gd-BOPTA; Bracco Diagnostics) maintain their recommendation for liver imaging, because they meet important diagnostic needs and comparable alternatives are missing [13, 14].

Various methods have been attempted to address issues related to GBCAs, including chelation therapy after image acquisition and AI-based synthetic contrast-enhanced imaging [4, 15–18]. These concerns also spurred the search for potentially safer alternatives, particularly those based on non-gadolinium elements, including manganese-based contrast agents, iron chelate-based contrast agents (IBCAs), and water proton chemical exchange dependent saturation transfer [19–24].

IBCAs have emerged as promising alternatives to GBCAs owing to the ubiquity of iron in body cells and the extensive systems for safe uptake, transport, and storage of iron. Superparamagnetic iron oxide nanoparticles have been explored for liver imaging because of their ability to be taken up by Kupffer cells in the reticuloendothelial system, resulting in their complete uptake into the body’s iron stores. However, the clinical use of superparamagnetic iron oxide nanoparticles has declined due to challenges related to stability, sensitivity, their less-favorable negative contrast, slow kinetics and safety [21, 25, 26]. To address these limitations, we investigated low-molecular-weight IBCAs that behave similarly to GBCAs and share rapid kinetics, including minimal retention in the body. In our previous studies [23, 27, 28], we demonstrated the potential of iron(III)-*trans*-cyclohexane diamine tetraacetic acid ([Fe(*t*CDTA)]^-^) and its derivatives for T1 dynamic contrast-enhanced (DCE) MRI and ratiometric pH-responsive MRI. In particular, iron(III)-*trans-1,4-diaminocyclohexane-t*CDTA-dimer demonstrated higher kinetic stability and T1 relaxivity than [Fe(*t*CDTA)]^-^, exhibiting a comparable relative enhancement (RE) to that of gadobutrol ([Gd-DO3A-butrol], Gadovist®, Bayer HealthCare Pharmaceuticals, Germany) at the same molecular dose in blood [28]. These results encouraged further exploration of [Fe(*t*CDTA)]^-^ derivatives for liver-targeted MRI.

In this study, we explored the derivative iron(III)-4-Ethoxybenzylamine-*t*CDTA ([Fe-EOB-*t*CDTA]). The design of [Fe-EOB-*t*CDTA] leverages the hepatocyte-specific targeting properties of the ethoxybenzyl moiety, similar to [Gd-EOB-DTPA]^2^^-^, while utilizing iron as the contrast-generating element to provide a potential alternative for liver MRI, particularly in patients with contraindications to GBCAs.

## Methods

### Synthesis of Fe-EOB-tCDTA

**Fig. 1:**
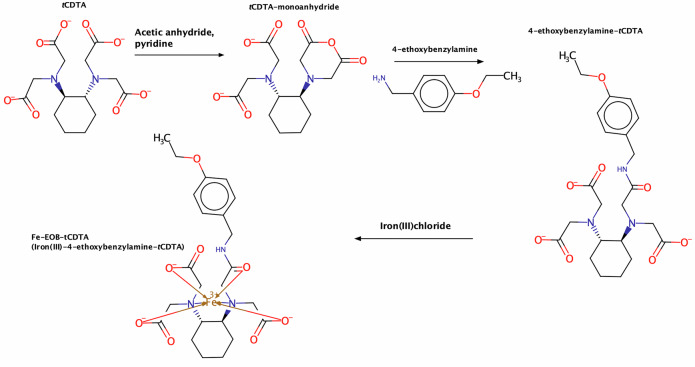
Synthesis of [Fe-EOB-tCDTA]. First, the monoanhydride of tCDTA was generated. Subsequently, 4-ethoxybenzylamine was combined with tCDTA-MA to produce EOB-tCDTA. Finally, EOB-tCDTA and FeCl₃ were chelated to produce [Fe-EOB-tCDTA] chelate

The synthesis was performed in three steps (Fig. 1). First, *trans-1,2-diaminocyclohexane-N, N, N’, N’-tetraacetic acid* monoanhydride (*t*CDTA-MA) was synthesized by stirring *trans-1,2-diaminocyclohexane*-N, N, N′, N′-tetraacetic acid (*t*CDTA, ≥ 99%, Carl Roth GmbH, Karlsruhe, Germany) with acetic anhydride (≥ 99%, Carl Roth GmbH) and pyridine (≥ 99%, Carl Roth GmbH) for 24 h under argon; the products was then washed with additional acetic anhydride (≥ 99%, Carl Roth GmbH), followed by ethyl acetate (≥ 99.5%, Carl Roth GmbH) [29]. Second, 2 g (5.77 mmol) of *t*CDTA-MA was added to a solution of 10.3 mL of dimethyl sulfoxide (DMSO, ≥ 99.5%, Carl Roth GmbH), followed by the addition of 0.96 g (6.35 mmol) of 4-ethoxybenzylamine (4-Ethoxyphenylmethanamine, ≥ 95%, BLDPharmtech Ltd., Shanghai, China). The mixture was stirred overnight under argon atmosphere to yield 4-ethoxybenzylamine-*t*CDTA (EOB-*t*CDTA). Dimethyl sulfoxide was then removed from the products by rotary evaporation, and the products were washed four times with ether. The resulting solid was isolated by recrystallization from the water. Subsequently, EOB-*t*CDTA was chelated with FeCl_3_ solution (0.3 mol/L) at a molar ratio of 1:1.01, and the mixture was stirred for 2 h to yield iron (III) 4-ethoxybenzylamine-*t*CDTA (Fe-EOB-*t*CDTA). After synthesis, the pH was adjusted to 7.4 using meglumine. The iron (III) hydroxide formed from excess FeCl_3_ during the procedure was filtered using syringe filters (0.22 μm, Rotilabo, Carl Roth GmbH, Karlsruhe, Germany) after centrifugation (1,000 rpm, 5 min, 3×). Following filtration, [Fe-EOB-*t*CDTA] was autoclaved at 121 °C and 15 psi for 15 min. Chemical structures were drawn using MarvinSketch software (https://www.chemaxon.com).

### Mass spectrometry analysis

After purification, MALDI mass spectrometry (Microflex LRF, Bruker) was used to verify the EOB-*t*CDTA. The measurement was performed in the positive reflector mode with a laser repetition rate of 60 Hz and lens voltage of 8.7 kV. The matrix used was *a*-cyano-4-hydroxycinnamic acid.

### High-performance liquid chromatography

EOB-*t*CDTA and [Fe-EOB-*t*CDTA] were identified using reverse-phase high-performance liquid chromatography (HPLC, DIONEX ULtiMate 3,000 system, NUCLEOSIL C18 column) following autoclaving. The compounds were diluted in H_2_O to a concentration of 1–2 mM. The gradient program was as follows: 2–66% acetonitrile with 5% ammonium bicarbonate at pH 7.8.

### *In vitro* characterization

### Relaxivity measurement

Relaxivity measurements were performed using a 1.41-T NMR spectrometer (MQ 60 Minispec, Bruker) and a 3-T MAGNETOM Lumina scanner (Siemens) to evaluate the magnetic field and medium dependence of the T1 relaxivity of [Fe-EOB-*t*CDTA] and the difference between [Fe-EOB-*t*CDTA] and [Gd-EOB-DTPA]^2^^-^. All compounds were diluted in water or fetal bovine serum (Gibco, Thermo Fisher Scientific) to three different concentrations (0.5, 1, and 2 mM, three duplicates). Measurements were performed at 37 °C, 1.41 T/23 °C, 3 T. For the 3-T measurement, standard two-dimensional spin-echo sequences were applied with the following parameter settings: repetition times = 100, 150, 300, 600, and 1,000 ms; echo time = 13 ms; matrix size = 256 × 256; and field of view = 75 × 75 mm^2^.

### Evaluation of kinetic complex stability by transmetalation measurements

The stability of [Fe-EOB-*t*CDTA] and Fe-*t*CDTA was assessed by measuring the changes in the absorption spectra between 380 and 680 nm over time during the zinc-stress test. According to Laurent et al [30]. In the zinc stress test, samples were prepared as follows: iron complexes (2.5 mM) were mixed with an equivalent of zinc chloride in sodium phosphate buffer solution (pH = 7, Na_2_HPO_4_ = 41 mM). The iron(III) released from the chelate forms insoluble iron phosphate, which precipitates out of the measured solution. Instead of relaxivity measurements, spectrometric measurements were performed at 23 °C for 9 h using a spectrometer (SPECORD 205, Analytik Jena). Given the slight solubility of gadolinium phosphate (solubility product constant ranging from 2.07 × 10⁻¹⁴ to 4.76 × 10⁻¹³) [30, 31], its application in observing the stability and transmetalation of GBCA is therefore limited to less stable chelates. In contrast, iron phosphate exhibits an extremely low solubility (solubility product constant ranging from 1 × 10⁻²⁶ to 1.3 × 10⁻²²), making it well-suited for the study of iron complexes, even with high stabilities [27]. The decrease in light absorption of iron chelates due to iron phosphate precipitation was measured at a wavelength of 410 nm. Relative absorption = (Abs/Abs_baseline_) × 100%.

### Cytotoxicity evaluation

BRL-3A cells (ATCC, Manassas, VA, USA) were grown under the following conditions: DMEM/F12 medium containing GlutaMAX, 10% fetal bovine serum, 1,000 U/mL penicillin, and 100 μg/mL streptomycin (Gibco, Thermo Fisher Scientific) at 37 °C with 5% CO₂. Cells between passages 2 and 8 were used for the cytotoxicity assay at a seeding density of 10⁴ cells per well in a 96-well plate (Falcon). The 3-(4,5-dimethylthiazol-2-yl)-2,5-diphenyltetrazolium bromide (MTT) assay (ab211091; Abcam) was used to evaluate cytotoxicity. Briefly, after reaching 80% confluence, the cells were treated with [Gd-EOB-DTPA]^2^^-^, [Fe-EOB-*t*CDTA], and carbonyl cyanide m-chlorophenyl hydrazone (used as a positive control to validate MTT reagent). The cells were then incubated at 37 °C and 5% CO₂ for 2 h at different final concentrations (1, 2.5, 5, 10, and 20 mM). After incubation, the culture medium was removed, and 50 μL of serum-free culture medium and 50 μL of MTT reagent were added to each well. The plates were incubated for 3 h. Next, 150 μL of MTT buffer was added, and the plate was placed on a shaker for 15 min. Absorbance was measured at 590 nm using a microplate reader (Power Wave XS2, BioTek). Cell viability (%) was calculated as follows: Abs_treated_/Abs_blank_ × 100%.

### Laser ablation–inductively coupled plasma–mass spectrometry (LA-ICP-MS)

BRL-3A cells were seeded and incubated in 8-well chamber slides (Nunc Lab-Tek II CC2; Thermo Fisher Scientific). After reaching 80% confluence, the cells were treated with [Gd-EOB-DTPA]^2^^-^ and [Fe-EOB-*t*CDTA] at the following ratios: blank, [Gd-EOB-DTPA]^2^^-^, and [Gd-EOB-DTPA]^2^^-^: [Fe-EOB-*t*CDTA] at ratios of 1:0.5, 1:1, 1:2.5, 1:5, 1:10, and 1:20 (mM). The slides were then washed with PBS (three times) to remove surface remnants after 15 min of incubation. The slide was dried on the hood. Laser ablation was performed using an NWRimage laser ablation system (266 nm, Elemental Scientific Laser, Bozeman, MT, USA) equipped with a low-dispersion ablation cell in a TwoVol3 ablation chamber attached to an ICP-ToF-MS (Model 2 R, Tofwerk AG, Thun, Switzerland) using a 0.762 mm ID PEEK tubing and a Dual Concentric Injector (DCI2). A square laser beam of 5 × 5 μm was used, and the samples were scanned with a repetition rate of 100 Hz and a fluence of 3 J/cm^2^ [32, 33]. Data were reduced using the Iolite v4 software (Elemental Scientific Laser). Further details of the analytical routine are described in Schannor et al [34].

### *In vivo* 3-T MRI

In the Animal Facility of Charité Universitätsmedizin Berlin, adult Balb/c mice weighing approximately 23–25 g (Janvier Labs) were housed under optimized environmental conditions: 12-h light/dark cycle, temperature of ~20 °C, and 40‒60% humidity. For each acquisition, the mice were anesthetized with isoflurane (1–2 vol% in oxygen) and kept on a temperature-controlled waterbed. First, the mice were imaged using a 2D T1-weighted fast low-angle shot (FLASH) sequence before administration of the contrast agent. Then, an intravenous dose of 0.2 or 0.3 mmol/kg of [Fe-EOB-*t*CDTA] or 0.025 mmol/kg body weight of [Gd-EOB-DTPA]^2^^-^ was administered through a tail vein catheter, followed by 100 μL of heparin saline (1,000 USP units/mL) to flush the catheter. One minute after injection, DCE images were acquired using a 3-T MAGNETOM Lumina scanner with the three-dimensional T1-weighted FLASH sequence and the following parameters: repetition time = 8.7 ms; echo time = 3.1 ms; matrix size = 256 × 256; flip angle = 20°; transmit/receive configuration = 2; slice thickness = 1 mm; number of repetitions = 28; acquisition time = 46:50 min:s. The animal experiments were approved by the LAGeSo Berlin (animal project number: G0067/19).

### Image analysis

Images were analyzed using Horos software (Horos Project, Switzerland) and Fiji software (ImageJ, NIH). All regions of interest (ROIs) were manually drawn by one board-certified radiologist. A second senior radiologist independently reviewed and verified the ROI positions to ensure accuracy and consistency. The ROIs were placed in predefined anatomical sites on each slide: the cerebrum, cardiac left ventricular lumen, liver segments V, VII, or VIII, renal cortex lower pole, and vastus medialis muscle. The ROI area was ≥ 2 mm². Relative enhancement (RE%) = [(SI_*post*_ - SI_*pre*_)/SI_*pre*_] × 100%, where SI = signal intensity.

### Statistical analysis

Results are presented as mean ± standard error. The T1 relaxivity and MRI results were analyzed using Two-way and One-way analysis of variance with *post hoc* Tukey’s multiple comparisons (all-*versus*-all comparisons) or Dunnett’s multiple comparisons. An unpaired *t*-test was performed to compare the RA%. A multi-unpaired *t*-test was performed to assess cell viability (%). Data were processed using Prism 10.0 (GraphPad Software, Inc.), and statistical significance was defined as *p* < 0.05. Significant differences between groups are denoted as follows: * *p* < 0.05, ** *p* < 0.01, *** *p* < 0.001, and **** *p* < 0.0001.

## Results

### Properties of EOB-tCDTA and Fe-EOB-tCDTA

The compound was synthesized according to a literature protocol with one slight modification regarding the solvent: DMSO was used instead of DMF [35]. The purity of EOB-*t*CDTA was controlled using HPLC after synthesis and purification (Supplemental Fig. S1). The retention time of EOB-*t*CDTA was 8.49 min. The product identities were confirmed by MALDI-MS (Supplemental Fig. S2). After the formation of the iron complex Fe-EOB-*t*CDTA, heat autoclaving and HPLC were performed. In contrast to [Fe-(*t*CDTA)]^-^, no precipitate was formed by autoclaving Fe-EOB-*t*CDTA, which supports the high thermal stability of this complex. The retention times of [Fe-EOB-*t*CDTA] before and after sterilization were 2.13 min and 2.26 min, respectively (Supplemental Fig. S1).

#### T1 relaxivities in water and serum

**Fig. 2:**
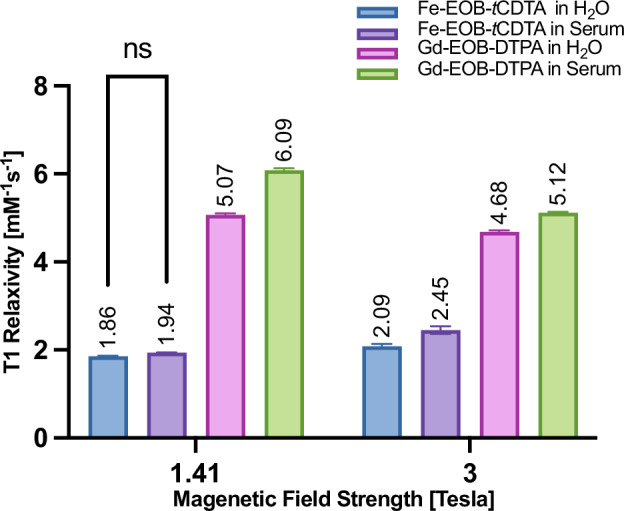
T1 relaxivity of [Fe-EOB-tCDTA] and [Gd-EOB-DTPA]^2^^-^. Measurements were performed in water and fetal bovine serum at 1.41 and 3 T (three replicates, mean ± standard error). Statistical analysis was performed using two-way ANOVA with *post hoc* Tukey’s multiple comparisons. A statistically significant difference was observed for all data points, with the exception indicated with ns

T1 relaxivities of [Fe-EOB-*t*CDTA] and [Gd-EOB-DTPA]^2^^-^ are shown in Fig. 2. The *r*₁ values varied significantly depending on the contrast agent and testing conditions. [Gd-EOB-DTPA]^2^^-^ had significantly higher *r*₁ values than [Fe-EOB-*t*CDTA] under all tested conditions (*p* < 0.0001). In the serum, both exhibited higher *r*₁ values than in water at 3 T. In both media, [Fe-EOB-*t*CDTA] demonstrated higher *r*₁ values at 3 T than at 1.41 T, whereas [Gd-EOB-DTPA]^2^^-^ exhibited the opposite trend.

#### Kinetic stability comparison by transmetalation measurement of [Fe-EOB-tCDTA] and [Fe-(tCDTA)]

**Fig. 3:**
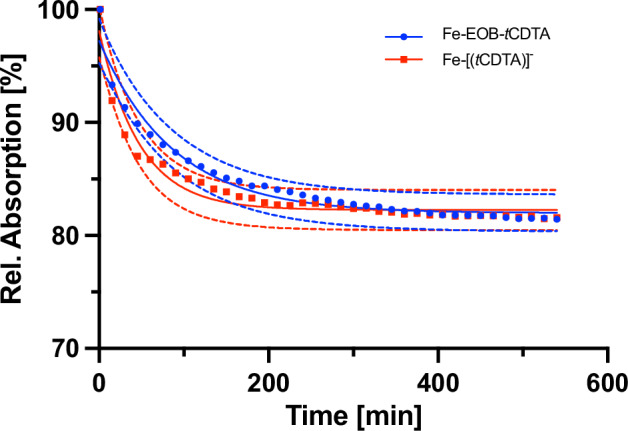
Evolution of [Fe-EOB-tCDTA] and [Fe-(tCDTA)]^-^ equilibrium under ZnCl_2_ challenge for 9 h. The released iron(III) from the iron complex is trapped by sodium phosphate, forming precipitating iron(III) phosphate. The relative absorbance of the remaining iron complexes was measured at 410 nm for 9 h (*n* = 3, with 95% prediction bands)

The light absorption spectra of [Fe-EOB-*t*CDTA] and [Fe-(*t*CDTA)]^-^ were obtained over a 9-h measurement (Supplemental Fig. S4). The evolution of absorption plateaus at 410 nm, which represents the equilibrium between the iron chelates and the precipitating iron phosphate (Fig. 3). At 540 min, the relative absorption of [Fe-EOB-*t*CDTA] and [Fe-(*t*CDTA)]^-^ approached the calculated equilibria of 81.98% (95% CI 81.50 to 82.24) and 82.26% (95% CI 81.87 to 82.63), respectively. The rate constants were 0.01144 (95% CI 0.009529 to 0.01366) and 0.02103 (95% CI 0.01667 to 0.02666), respectively.

#### Cytotoxicity examination of [Fe-EOB-tCDTA] and [Gd-EOB-DTPA]^2^^-^

**Fig. 4:**
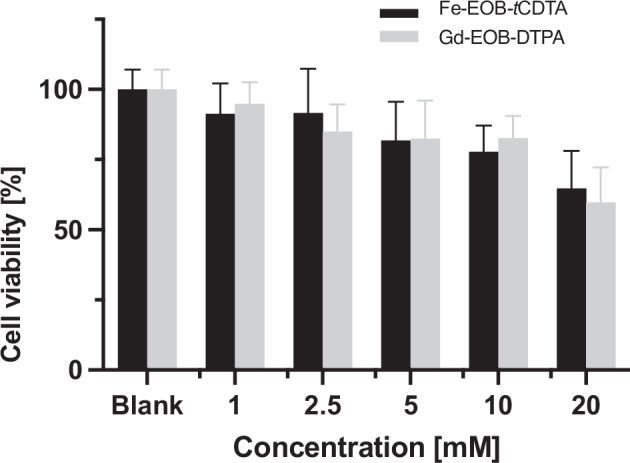
Toxicity of [Fe-EOB-tCDTA] and [Gd-EOB-DTPA]^2^^-^ in BRL-3A cells. The cells were exposed to different concentrations of contrast agents (0, 1, 2.5, 5, 10, and 20 mM) for 2 h. Viability was determined using the MTT assay after incubation (three replicates, *n* = 9, mean ± standard error). Statistical analyses were performed using multiple unpaired *t*-tests. No significant differences were observed between the various concentration groups

After 2 h of exposure to [Fe-EOB-tCDTA] and [Gd-EOB-DTPA]^2^^-^, cell viability was measured using the MTT assay (Fig. 4). Compared with the untreated controls, even at a concentration of 5 mM, [Fe-EOB-*t*CDTA] and [Gd-EOB-DTPA]^2^^-^ showed cell viabilities of 81.85 ± 4.56% and 82.43 ± 4.53%, respectively. No significant differences were observed between the various concentration groups.

#### *In vitro* inhibition of [Gd-EOB-DTPA]^2-^ uptake by [Fe-EOB-tCDTA] in LA-ICP-MS

**Fig. 5:**
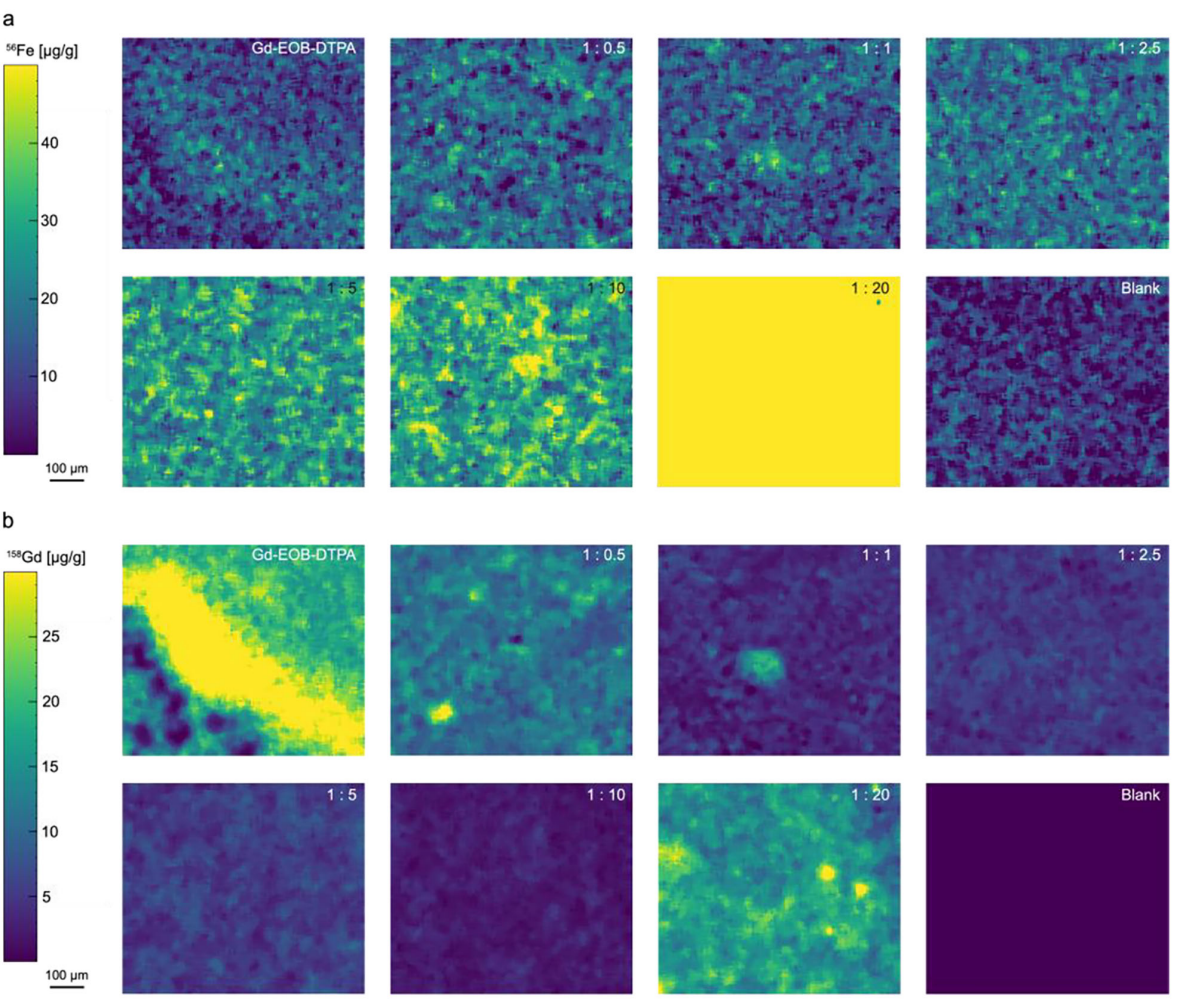
Laser ablation–inductively coupled plasma–mass spectrometry of [Fe-EOB-*t*CDTA] and [Gd-EOB-DTPA]^2^^-^ in BRL-3A cells. **a **^56^Fe mass spectrometry signal of endogenous iron and of [Fe-EOB-*t*CDTA]; **b **^158^Gd mass spectrometry signal of [Gd-EOB-DTPA]^-2^. The BRL-3A cells were treated with [Gd-EOB-DTPA]^2^^-^ only (1 mM) or with [Gd-EOB-DTPA]^2^^-^ and [Fe-EOB-*t*CDTA] at different molar ratios (1:0.5, 1:1, 1:2.5, 1:5, 1:10, and 1:20). Blank: No metal complexes.

BRL-3A cells were incubated with [Gd-EOB-DTPA]^2^^-^ (1 mM Gd) and increasing ratios/concentrations of [Fe-EOB-*t*CDTA] to investigate whether both substances compete for similar uptake pathways. Iron and gadolinium maps in BRL-3A cells were determined by LA-ICP-ToF-MS, and the results are shown in Fig. 5, with the corresponding microscopy images shown in the Supplemental Fig. S3. The iron maps reveal no substantial increase to the natural cellular iron content up to a concentration of 2.5 mM Fe (1:2.5). With a further increase in [Fe-EOB-*t*CDTA] (5- and 10-mM Fe), the cellular iron content increased, and with a ratio of 1:20 (20 mM Fe), the uptake was very high, corresponding to the increased toxicity of both [Gd-EOB-DTPA]^2^^-^ and [Fe-EOB-*t*CDTA] at this concentration (Fig. 4). Complementarily, the Gd images revealed a decrease in cellular Gd content with increasing ratios of [Fe-EOB-*t*CDTA] up to a ratio of 1:10, consistent with the replacement of [Gd-EOB-DTPA]^2^^-^ by Fe-EOB-*t*CDTA. Corresponding to the iron channel, the Gd map of the ratio 1:20 displayed a high Gd concentration relative to the other ratios, which might be a consequence of leaky cell membranes at these high concentrations (Fig. 4).

### Comparison of DCE-MRI properties in mouse

**Fig. 6:**
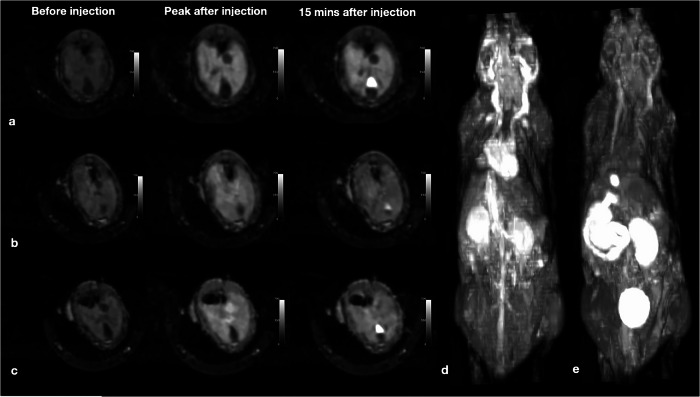
*In vivo* liver contrast effect on T1-weighted MRI in mice. Intravenous application of 0.025 mmol/kg [Gd-EOB-DTPA]^2^^-^ (**a**), 0.2 mmol/kg (**b**)/0.3 mmol/kg (**c**) [Fe-EOB-tCDTA] at 3 T. Left column: Sagittal slices through the liver and gallbladder before injection; middle column: 5 min for [Gd-EOB-DTPA]^2^^-^ or 1 min for [Fe-EOB-tCDTA] after injection; right column: 15 min after injection. Arterial phase (**d**) and biliary phase (**e**) of mice at maximum intensity projection. Three-dimensional T1-weighted FLASH sequence using a 3-T MANGETOM Lumina scanner. Window level/window width: 350/700. FLASH, Fast low-angle shot

DCE-MRI was performed after the administration of [Fe-EOB-*t*CDTA] (*n* = 6, both injection-dose groups) and [Gd-EOB-DTPA]^2^^-^ (*n* = 6). No adverse reactions affecting the well-being of the animals were observed during the examination or within 7 days after contrast agent administration. Figure 6 shows T1-weighted images of mice liver before and after contrast agent administration. Both contrast agents exhibited liver enhancement and gallbladder-excretion.

**Fig. 7:**
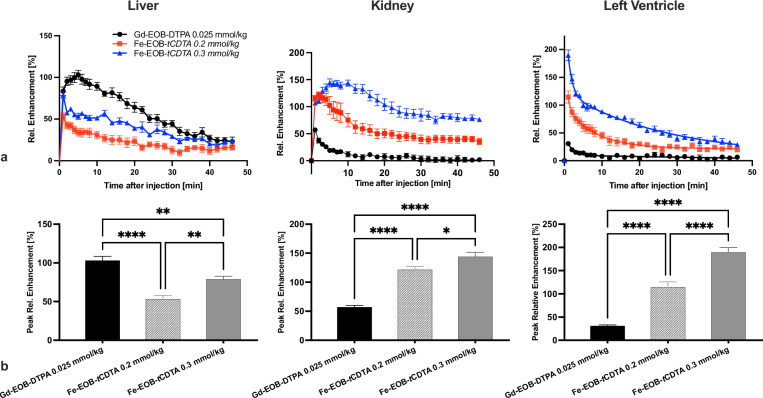
T1-weighted contrast enhancement time curves of the mice liver, kidney, and left ventricle of [Fe-EOB-tCDTA] compared with [Gd-EOB-DTPA]^2^^-^. **a** Relative signal enhancement time curves after intravenous injection of contrast agents. **b** Comparison of relative peak enhancement of mice after contrast agent injection, with significance indicated. Statistical analysis was performed using one-way ANOVA with *post hoc* Tukey’s multiple comparisons. ANOVA, Analysis of variance

The relative signal enhancement time curves are depicted in Fig. 7a, while Fig. 7b shows the peak enhancement of the liver, left ventricle, and kidneys of the mice. The pharmacokinetic properties of the chelates were evaluated based on three factors: time-to-peak (TTP), washout slope, and area under the curve (AUC) after injection (Table 1). In the liver, [Gd-EOB-DTPA]^2^^-^ demonstrated superior hepatocellular signal enhancement, achieving a peak RE% of 104.0 ± 3.7%, which was significantly higher than that of [Fe-EOB-*t*CDTA] at both doses (*p* = 0.0055 and *p* < 0.0001, compared to [Fe-EOB-*t*CDTA] at 0.3 mmol/kg and 0.2 mmol/kg, respectively). [Fe-EOB-*t*CDTA] exhibited dose-dependent liver signal, with the 0.3 mmol/kg dose yielding a 49% greater AUC_0-46_ than the 0.2 mmol/kg dose (*p* < 0.001). The washout kinetics were slowest for [Gd-EOB-DTPA]^2^^-^ (*p* = 0.02 and *p* < 0.001 for [Fe-EOB-*t*CDTA] at 0.3 mmol/kg and 0.2 mmol/kg, respectively), consistent with its hepatocellular specificity. Compared to [Gd-DO3A-butrol] at 0.1 mmol/kg (RE% = 131.0 ± 8.98, AUC_0-46_ = 1,600 ± 44.74, washout slope = -0.81 ± 0.04, Supplemental Fig. S5a, b) obtained in our previous study [27], [Fe-EOB-*t*CDTA] at 0.3 mmol/kg exhibited slower washout kinetics and liver retention (*p* < 0.001).

**Table 1:** DCE-MRI properties of [Fe-EOB-tCDTA] and [Gd-EOB-DTPA]^2^^-^ in the liver, kidney, and left ventricle

Parameter	Gd-EOB-DTPA 0.025 mmol/kg	Fe-EOB*-t*CDTA 0.2 mmol/kg	Fe-EOB*-t*CDTA 0.3 mmol/kg
SI predose (SI_*pre*_)	266.16 ± 6.17/246.67 ± 6.19/213.17 ± 4.69	266.79 ± 6.33/257.6 ± 6.6/207.0 ± 4.23	267.09 ± 5.61/278.9 ± 1.82/212.8 ± 4.37
SI postdose (SI_*post*_)	488.15 ± 13.85/387.3 ± 7.05/279.03 ± 5.13	408.76 ± 12.21/554.95 ± 13.67/444.48 ± 22.82	478.06 ± 9.93/577.28 ± 9.95/616.67 ± 20.9
Peak RE (%)	104.04 ± 3.73/57.18 ± 1.82/31.03 ± 1.70	53.13 ± 1.75/129.16 ± 8.64/114.37 ± 8.67	79.39 ± 13.14/153.60 ± 6.09/189.72 ± 7.02
TTP (min)	5/1/1	1/2.33 ± 0.56/1	1/8.67 ± 0.88/1
AUC_0-46_ (%·min)	2,669 ± 76.04/427.4 ± 73.3/373.9 ± 54.95	1,010 ± 68/2,629 ± 144/1,604 ± 81.05	1,762 ± 50.33/4,704 ± 127.4/2,983 ± 74.34
Washout slope (%/min)	-2.03 ± 0.15/-0.70 ± 0.06/-0.28 ± 0.02	-0.68 ± 0.04/-1.73 ± 0.22/-1.35 ± 0.18	-1.02 ± 0.03/-1.74 ± 0.14/-2.43 ± 0.17
K_Fast_ (min^-1^)	0.586 (0.289 to 0.882)	0.280 (0.186 to 0.374)	0.822 (0.602 to 1.04)
K_Slow_ (min^-1^)	0.0158 (0.0041 to 0.0276)	0.0215 (0.0132 to 0.0297)	0.0317 (0.0293 to 0.0342)

K_Fast_ and K_Slow_ are rate constants of the two-phase exponential decay fit of left ventricular REs (with 95% confidence intervals)

Data for liver/kidney/left ventricle are mean ± standard error

*AUC*_*0-46*_ Area under the curve from 0 to 46 min, *RE* Relative enhancement, *SI postdose* Peak signal intensity after injection, *TTP* Time to peak

[Fe-EOB-*t*CDTA] demonstrated stronger renal enhancement than [Gd-EOB-DTPA]^2^^-^. At 0.3 mmol/kg, [Fe-EOB-*t*CDTA] reached a peak RE% of 153.6 ± 6.1%, nearly triple that observed with Gd-EOB-DTPA (*p* < 0.0001). The TTP for [Fe-EOB-*t*CDTA] (0.3 mmol/kg) was delayed to 8.7 ± 0.9 min in the kidney, suggesting prolonged kidney retention, whereas [Gd-EOB-DTPA]^2^^-^ was cleared rapidly (TTP: 1 min). This was reflected in the AUC_0–46_, where [Fe-EOB-*t*CDTA] (0.3 mmol/kg) resulted in a 10-fold higher exposure than Gd-EOB-DTPA. Compared with [Gd-DO3A-butrol] at 0.1 mmol/kg (RE% = 126.62 ± 7.25, AUC_0-46_ = 2,178 ± 90.2, TTP = 1.5 ± 0.29 min), [Fe-EOB-*t*CDTA] showed similar peak RE% at both doses (*p* = 0.887 and *p* = 0.1553 for [Fe-EOB-tCDTA] at 0.2 mmol/kg and 0.3 mmol/kg, respectively), and [Fe-EOB-tCDTA]at 0.3 mmol/kg exhibited higher exposure.

In the left ventricle, [Fe-EOB-*t*CDTA] demonstrated a significantly higher blood peak RE% at both doses than [Gd-EOB-DTPA]^2^^-^ (*p* < 0.0001). The AUC_0-46_ for [Fe-EOB-*t*CDTA] (both doses) was higher than that for [Gd-EOB-DTPA]^2^^-^ (*p* < 0.0001). Compared to [Gd-DO3A-butrol] at 0.1 mmol/kg (131.0 ± 8.98, Supplemental Fig. S5), [Fe-EOB-*t*CDTA] at 0.3 mmol/kg demonstrated a substantially higher peak RE% (*p* = 0.0018) and at 0.2 mmol/kg, a similar peak RE% (*p* = 0.62) (Supplemental Fig. S5). Additionally, the AUC_0-46_ of [Fe-EOB-*t*CDTA] at 0.2 mmol/kg was similar to that of [Gd-DO3A-butrol] at 0.1 mmol/kg (AUC_0-46_ = 1,600 ± 44.74, Supplemental Fig. S5). Furthermore, a comparison of the washout slope of [Fe-EOB-*t*CDTA] at 0.3 mmol/kg with that of [Gd-DO3A-butrol] at 0.1 mmol/kg revealed a slower rate of decline (washout slope = -2.06 ± 0.06). In addition, a two-phase exponential decay fit of the left ventricular relative enhancements was done to summarize the pharmacokinetics of the chelates in blood (Table 1).

**Fig. 8:**
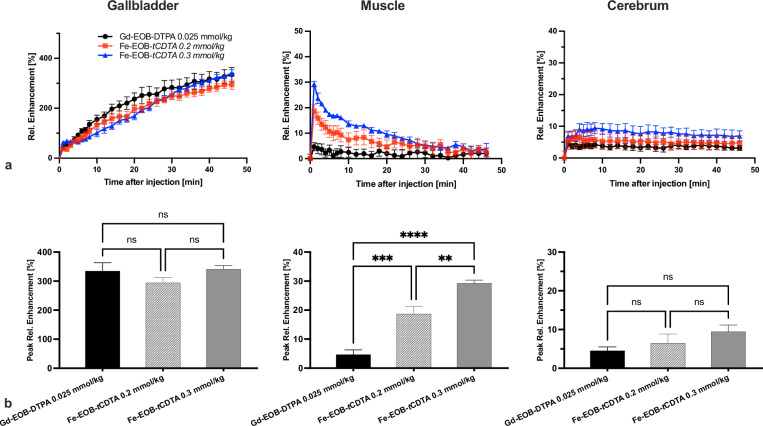
T1-weighted contrast enhancement time curves of the mice gallbladder, muscle, and cerebrum of [Fe-EOB-tCDTA] compared with [Gd-EOB-DTPA]^2^^-^. **a** Relative signal enhancement time curves after intravenous injection of the contrast agents. **b** Comparison of relative peak enhancement after contrast agent injection, with significance indicated. Statistical analysis was performed using one-way ANOVA with *post hoc* Tukey’s multiple comparisons. ANOVA, Analysis of variance

Figure 8 shows the RE% DCE time course and peak RE% in the gallbladder, muscle, and cerebrum after contrast agent administration. DCE-MRI properties are shown in Supplemental Table S1. In muscle, [Fe-EOB-*t*CDTA] showed a higher peak RE% than [Gd-EOB-DTPA]^2^^-^ at both doses (*p* = 0.001 for 0.2 mmol/kg and *p* < 0.0001 for 0.3 mmol/kg). In addition, [Fe-EOB-*t*CDTA] exhibited dose-dependent enhancement between the two doses (*p* = 0.0028). However, there was no significant difference compared to [Gd-DO3A-butrol] (Supplemental Fig. S5). In the gallbladder and cerebrum, no significant intergroup differences were observed in the peak RE%. The AUC_0-46_ analysis revealed significant intergroup differences. In muscle, [Fe-EOB-tCDTA] showed a dose-response (*p* = 0.007); both [Fe-EOB-*t*CDTA] doses were significantly higher than [Gd-EOB-DTPA]^2^^-^ (*p* = 0.003 and *p* < 0.001, for [Fe-EOB-*t*CDTA] at 0.3 mmol/kg and 0.2 mmol/kg, respectively). In the gallbladder, [Gd-EOB-DTPA]^2^^-^ exhibited the highest AUC_0-46_, which was significantly greater than both [Fe-EOB-*t*CDTA] doses (*p* < 0.001 for both doses). In the brain, [Fe-EOB-*t*CDTA] demonstrated substantial dose dependence (0.2 mmol/kg *versus* 0.3 mmol/kg: *p* < 0.001). In addition, [Fe-EOB-*t*CDTA] at both doses showed higher AUC_0-46_ than [Gd-EOB-DTPA]^2^^-^ (*p* = 0.009 and *p* < 0.001, for [Fe-EOB-*t*CDTA] at 0.2 mmol/kg and 0.3 mmol/kg, respectively).

## Discussion

In this study, we synthesized the [Fe-EOB-*t*CDTA] contrast agent, compared its stability with that of [Fe-(*t*CDTA)]^-^, and compared its relaxivity, cytotoxicity, and T1 enhancement with those of Gd-EOB-DTPA. Furthermore, T1 enhancement in the liver and blood samples of the [Fe-EOB-*t*CDTA] and [Gd-EOB-DTPA]^2^^-^ groups was compared with that of the [Gd-DO3A-butrol] group. Our findings suggest that [Fe-EOB-*t*CDTA] is taken up as [Gd-EOB-DTPA]^2^^-^ by functional hepatocytes and is subsequently excreted in the bile, accumulating in the gallbladder and intestines. However, the required injection dose, mechanism of cellular uptake, excretion through the biliary pathway, and imaging characteristics of these two compounds vary. In addition, [Fe-EOB-*t*CDTA] demonstrated comparable RE% in blood to that of [Gd-DO3A-butrol] (0.1 mmol/kg) at a doubled injection dose of 0.2 mmol/kg. These findings suggest that [Fe-EOB-*t*CDTA] may be a suitable T1 MR contrast agent with good blood contrast and biliary excretion, although it cannot reproduce the strong and long-lasting liver contrast of [Gd-EOB-DTPA]^2^^-^.

The *in vitro* examination of the T1 relaxivity of [Fe-EOB-*t*CDTA] and [Gd-EOB-DTPA]^2^^-^ in water and serum in this study demonstrated that [Fe-EOB-*t*CDTA] has a lower T1 relaxivity. This is consistent with the results of previous studies showing gadolinium’s superior T₁-shortening capability due to its seven unpaired electrons (*versus* five of Fe³⁺) and faster water-exchange kinetics [36–39]. However, [Fe-EOB-*t*CDTA] exhibited higher *r₁* values at 3 T than at 1.41 T, which has the opposite trend to that of [Gd-EOB-DTPA]^2^^-^ and reduces the gap at the common field strength of 3 T.

While the instability of [Fe-(*t*CDTA)]^-^ during autoclaving presents a significant disadvantage for potential clinical translation [27], [Fe-EOB-*t*CDTA] exhibited stability during heat sterilization. To further investigate the potential differences in kinetic properties, the stability of IBCAs was examined using spectrometric measurements in the presence of zinc chloride and phosphate. Despite the similar thermodynamic stability indicated by the comparable plateau, [Fe-EOB-*t*CDTA] exhibited superior kinetic stability compared to [Fe-(*t*CDTA)]^-^, demonstrating higher resistance to transmetalation with zinc, which indicates that the ethoxybenzyl moiety in [Fe-EOB-tCDTA]stabilizes this iron complex. The reduced rate of iron release further reduces the risk of free iron-induced toxicity *in vivo*, a salient benefit when compared to less stable gadolinium agents (which release toxic Gd³⁺ upon transmetalation). However, even if trace amounts of Fe³⁺ ions are released, the human iron metabolism system has sufficient capacity to process them, as the human body stores approximately 4 g of required Fe³⁺ ions in comparison [23, 40]. This is indirectly supported by the uptake of increasing concentrations of [Fe-EOB-*t*CDTA] in the competition experiment (Fig. 5), which illustrates the substantial natural cellular iron content, which is not substantially increased up to a concentration of 2.5 mM in stark contrast to the uptake of [Gd-EOB-DTPA]^2^^-^ at 1 mM.

The MTT assay results demonstrated that both [Fe-EOB-*t*CDTA] and [Gd-EOB-DTPA]^2^^-^ exhibited good short-term cytocompatibility, with cell viabilities exceeding 80% even at high concentrations (5 mM) after 2 h of exposure, suggesting low acute cytotoxicity under the tested conditions, a prerequisite for the potential clinical translation of [Fe-EOB-*t*CDTA].

In MRI of mice, [Fe-EOB-*t*CDTA] and [Gd-EOB-DTPA]^2^^-^ exhibited different pharmacokinetic and signal enhancement properties in the liver. The results demonstrate that [Fe-EOB-*t*CDTA] shows promising dose-dependent liver signal enhancement and hepatobiliary excretion with favorable pharmacokinetic properties but lacks the strong and long-lasting hepatocellular signal enhancement characteristics of [Gd-EOB-DTPA]^2^^-^. The performance of [Gd-EOB-DTPA]^2^^-^ confirms its well-established hepatocellular specificity, which is mediated through efficient organic anion-transporting polypeptides (OATP)1B1/1B3 transporter uptake and subsequent biliary excretion [20, 41, 42]. However, the observed dose-dependent liver signal and biliary excretion of [Fe-EOB-*t*CDTA] suggest that this novel IBCA also demonstrates hepatocellular uptake, albeit through potentially different mechanisms. Proteomic analysis of OATP1B1/1B3 found that OATP1B1 accounted for 22% of the total targeted protein, whereas OATP1B3 was expressed at a significantly lower level (approximately 8%). OATP1B1 primarily transports organic anions (*e.g*., bilirubin, bile acids, and statins), whereas OATP1B3 also transports neutral molecules with amphipathic features [43–45]. In contrast to the double-negatively charged [Gd-EOB-DTPA]^2^^-^, the transport of the neutral [Fe-EOB-*t*CDTA] may be mainly mediated by OATP1B3, which may explain the relatively low liver enhancement. Alternatively, this could be mediated by OATP2B1 or MRP3, which transport neutral molecules [46, 47]. However, as the hepatic amount of the OATP1B transporter is approximately one order of magnitude higher than that of OATP2B1 [47], the hepatic uptake of [Fe-EOB-*t*CDTA] mediated by OATP2B1 is likely to be very limited.

McRay et al [35]. employed the same tCDTA-monoanhydride-based synthesis route as previously described by us [29] to synthesize EOB-*t*CDTA as in this study, but chelated manganese as a paramagnetic metal. Because Mn^2+^ is only double-charged in contrast to Fe^3+^, the overall charge of the Mn-EOB-*t*CDTA complex is single negative. Remarkably, this compound provided a similar T1 relaxivity as [Fe-EOB-*t*CDTA] but demonstrated a strong, long-lasting liver contrast comparable to [Gd-EOB-DTPA]^2^^-^ at a dose of 0.1 mmol Mn/kg, highlighting that the negative overall charge appears to be a fundamental precondition for the desired strong, long-lasting liver contrast imaging properties.

Likewise, Nyström et al [20]. reported that Mn(III)TriCP-PhOEt, which contains an ethoxybenzyl moiety as well as a negative charge, exhibited hepatocyte-specific OATP1 channel targeting *in vitro* and strong but delayed liver enhancement *in vivo*. The superior performance of Mn(III)TriCP-PhOEt in the liver compared to neutral [Fe-EOB-*t*CDTA] further confirms that the ethoxybenzyl side group facilitates hepatocyte uptake, whereas the anionic property is crucial for liver accumulation.

In the kidneys, [Fe-EOB-*t*CDTA] exhibited significantly higher and more protracted enhancement than [Gd-EOB-DTPA]^2^^-^, which is comparable to the RE% of [Gd-DO3A-butrol] [27]. This prolonged kidney retention could result from protein binding in the renal interstitium, active tubular reabsorption, or transient interactions with tubular transport systems. For instance, OATP2B1 is also expressed in the kidney and skeletal muscle [47], which may cause kidney and muscle accumulation and prolonged drug retention. Notably, [Fe-EOB-*t*CDTA] demonstrated a similar peak RE% at 0.2 mmol/kg as [Gd-DO3A-butrol] at 0.1 mmol/kg, but a slower washout slope, particularly when the concentration of [Fe-EOB-*t*CDTA] is increased. Some [Fe-EOB-*t*CDTA] remains in the blood for some time after the imaging procedure. However, it is unlikely to cause safety concerns since the risk of an increased free iron concentration in the blood seems low, as [Fe-(*t*CDTA)]^-^ has approximately 100,000-fold higher stability constants than the main carrier of plasma iron, transferrin, which constantly releases traces of free iron [27]. The renal enhancement observed with [Fe-EOB-*t*CDTA] is substantially longer than that observed with [Gd-EOB-DTPA]^2^^-^ and somewhat longer than that observed with [Gd-DO3A-butrol]. This difference may be partially caused by the higher doses of [Fe-EOB-*t*CDTA] and [Gd-DO3A-butrol] and may provide special opportunities for renal imaging, more detailed visualization or tumor characterization. The prolonged retention may not be associated with nephrotoxicity, as the safety of ferumoxytol, which has a similar prolonged kidney signal, has already been established in patients with chronic renal insufficiency [48, 49]. Other iron chelates have demonstrated physiological metabolism through endogenous iron pathways without tissue deposition or fibrosis [50]. However, further pharmacokinetic and toxicological studies are needed to clarify the underlying clearance mechanisms and ensure safety.

While the liver signal of [Fe-EOB-*t*CDTA] was inferior to that of [Gd-EOB-DTPA]^2^^-^, gallbladder accumulation was comparable, demonstrating partial hepatic excretion in addition to renal excretion. Although [Fe-EOB-*t*CDTA] inhibited the uptake of [Gd-EOB-DTPA]^2^^-^ in a liver cell line, the significantly higher enhancement in the kidney and strong signal in the bladder could indicate that the biliary excretion of [Fe-EOB-*t*CDTA] is lower than that of [Gd-EOB-DTPA]^2^^-^, which has a renal/biliary excretion ratio of approximately 1 [51]. Thus, [Fe-EOB-*t*CDTA] is a dual-excretion agent (renal + biliary) more similar to gadobenate dimeglumine (MultiHance) [52]. It may be better suited for combined renal and biliary imaging than specifically liver MRI or used in patients with renal insufficiency, which could be compensated by biliary excretion.

In conclusion, the attachment of the 4-ethoxybenzylamine moiety to *t*CDTA enabled the expected liver targeting and biliary excretion of [Fe-EOB-*t*CDTA] but could not provide the strong, long-lasting liver signal enhancement of [Gd-EOB-DTPA]^2^^-^. The comparison with Mn-EOB-*t*CDTA strongly suggests that at least one negative charge is required for the complexes to achieve high T1 contrast in the liver, for example, by transient receptor binding. However, compared to [Gd-DO3A-butrol], [Fe-EOB-*t*CDTA] demonstrated comparable blood enhancement at only a doubled injection dose with additional hepatobiliary excretion. These findings suggest that it may have potential as a nonspecific extracellular contrast agent without being a dedicated liver contrast agent.

## Supplementary information

**Additional file 1: Fig. S1** HPLC results for EOB-tCDTA (a) and Fe-EOB-tCDTA before (b) and after (c) autoclaving. Method: gradient reverse phase; mobile phase: 2-66% acetonitrile with ammonium bicarbonate at PH 7.8. Flow rate: 1.0 mL/min for 20 min. The retention time of EOB-tCDTA was 8.49 min and the main peak area was 98.26%. The retention times of Fe-EOB-tCDTA before and after sterilization were 2.13 and 2.26 min, respectively. **Fig. S2** MALDI Mass Spectrometry for EOB-tCDTA Expected mass: 479.56 g/mol. Theoretical masses: [EOB-tCDTA+H]+: 480.56 g/mol; [EOB-tCDTA+Na]+: 502.56 g/mol. **Fig. S3** Microscopic images taken before LA-ICP-MSI of Fe- EOB-tCDTA and Gd-EOB-DTPA in BRL-3A cells. BRL-3A cells were treated with Gd-EOB-DTPA only or with Gd-EOB-DTPA and Fe-EOB-tCDTA at different ratios (1:0.5, 1:1, 1:2.5, 1:5, 1:10, and 1:20). **Fig. S4** Spectral light absorption curves of Fe-EOB-tCDTA (a) and [Fe-(tCDTA)]-(b) during zinc challenge over 9 h. Absorption measurements were performed immediately after mixing with the [ZnCl2 Na2HPO4] solution. The same [ZnCl2 Na2HPO4] solution was used as a reference for all measurements. The wavelength of 410 nm was chosen for the time curves in Figure 3. **Fig. S5** T1 contrast enhancement of Fe-EOB-tCDTA and Gd-EOB-DTPA in mice compared with that of Gd-DO3A-butrol. (a) Relative signal enhancement time curves of the cardiac left ventricle, liver, kidney, and muscle after intravenous injection of contrast agents. (b) Comparison of peak relative enhancement in the cardiac left ventricle, liver, kidney, and muscle of mice after contrast agent injection, with significance indicated. Statistical analysis was performed using one-way ANOVA with Dunnett's multiple comparisons (Gd-DO3A-butrol as control). ANOVA, analysis of variance. **Table S1** DCE-MRI properties of Fe-EOB-tCDTA and Gd-EOB-DTPA in the gallbladder, muscle, and cerebrum.

## Data Availability

The datasets used and/or analyzed during the current study are available from the corresponding author on reasonable request.

## Abbreviations

AUC: Area under the curve
DCE: Dynamic contrast-enhanced
Fe-EOB-tCDTA: Iron(III)-4-Ethoxybenzylamine-tCDTA
FLASH: Fast low-angle shot
GBCAs: Gadolinium-based contrast agents
HPLC: High-performance liquid chromatography
IBCAs: Iron-based contrast agents
LA-ICP-MS: Laser ablation–inductively coupled plasma–mass spectrometry
MRI: Magnetic resonance imaging
MTT: 3-(4,5-dimethylthiazol-2-yl)-2,5-diphenyltetrazolium bromide
OATP: Organic anion-transporting polypeptide
ROI: Region of interest
*t*CDTA: Trans-1,2-diaminocyclohexane-N,N,N′,N′-tetraacetic acid
TTP: Time to peak

## References

[1] Wahsner J, Gale EM, Rodríguez-Rodríguez A, Caravan P (2019) Chemistry of MRI contrast agents: current challenges and new frontiers. Chem Rev 119:957–1057. 10.1021/acs.chemrev.8b00363 30350585 10.1021/acs.chemrev.8b00363 PMC6516866

[2] Weinreb JC, Rodby RA, Yee J et al (2021) Use of intravenous gadolinium-based contrast media in patients with kidney disease: consensus statements from the American College of Radiology and the National Kidney Foundation. Radiology 298:28–35. 10.1148/radiol.2020202903 33170103 10.1148/radiol.2020202903

[3] Ayers-Ringler J, McDonald JS, Connors MA et al (2022) Neurologic effects of gadolinium retention in the brain after gadolinium-based contrast agent administration. Radiology 302:676–683. 10.1148/radiol.210559 34931861 10.1148/radiol.210559 PMC8893178

[4] Semelka RC, Ramalho M (2023) Gadolinium deposition disease: current state of knowledge and expert opinion. Invest Radiol 58:523–529. 10.1097/RLI.0000000000000977 37058336 10.1097/RLI.0000000000000977

[5] Tweedle MF (2021) Gadolinium retention in human brain, bone, and skin. Radiology 300:570–571. 10.1148/radiol.2021210957 34128728 10.1148/radiol.2021210957

[6] Parillo M, Mallio CA, Van Der Molen AJ et al (2023) Skin toxicity after exposure to gadolinium-based contrast agents in normal renal function, using clinical approved ooses: current status of preclinical and clinical studies. Invest Radiol. 10.1097/RLI.0000000000000973 10.1097/RLI.0000000000000973 37185158

[7] Domingo JL, Semelka RC (2025) Gadolinium toxicity: mechanisms, clinical manifestations, and nanoparticle role. Arch Toxicol. 10.1007/s00204-025-04124-x 10.1007/s00204-025-04124-x PMC12454587 40608128

[8] Frenzel T, Apte C, Jost G et al (2017) Quantification and assessment of the chemical form of residual gadolinium in the brain after repeated administration of gadolinium-based contrast agents: comparative study in rats. Invest Radiol 52:396–404. 10.1097/RLI.0000000000000352 10.1097/RLI.0000000000000352 PMC5464750 28125438

[9] Jost G, Frenzel T, Boyken J et al (2019) Long-term excretion of gadolinium-based contrast agents: linear *versus* macrocyclic agents in an experimental rat model. Radiology 290:340–348. 10.1148/radiol.2018180135 30422091 10.1148/radiol.2018180135

[10] Runge VM (2018) Dechelation (transmetalation): consequences and safety concerns with the linear gadolinium-based contrast agents, in view of recent health care rulings by the EMA (Europe), FDA (United States), and PMDA (Japan). Invest Radiol 53:571–578. 10.1097/RLI.0000000000000507 30130320 10.1097/RLI.0000000000000507

[11] U.S. Food and Drug Administration (2018) FDA drug safety communication: FDA warns that gadolinium-based contrast agents (GBCAs) are retained in the body; requires new class warnings. Available via https://www.fda.gov/drugs/drug-safety-and-availability/fda-drug-safety-communication-fda-warns-gadolinium-based-contrast-agents-gbcas-are-retained-body. Accessed 21 Feb 2026

[12] Kloc M, Halasa M, Wosik J, Ghobrial RM (2025) Gadolinium-based MRI contrast agent effects on calcium signaling and actin-dependent cell functions. Magn Med 1:100004. 10.1016/j.magmed.2025.100004

[13] European Medicines Agency (2017) EMA’s final opinion confirms restrictions on use of linear gadolinium agents in body scans. Available via https://www.ema.europa.eu/en/news/emas-final-opinion-confirms-restrictions-use-linear-gadolinium-agents-body-scans. Accessed 21 Feb 2026

[14] Runge VM (2017) Critical questions regarding gadolinium deposition in the brain and body after injections of the gadolinium-based contrast agents, safety, and clinical recommendations in consideration of the EMA’s pharmacovigilance and risk assessment committee recommendation for suspension of the marketing authorizations for 4 linear agents. Invest Radiol 52:7 10.1097/RLI.0000000000000374 28368880

[15] Boyken J, Frenzel T, Lohrke J et al (2019) Impact of treatment with chelating agents depends on the stability of administered GBCAs: a comparative study in rats. Invest Radiol 54:76–82. 10.1097/RLI.0000000000000522 30358694 10.1097/RLI.0000000000000522 PMC6310454

[16] Layne KA, Wood DM, Dargan PI (2020) Gadolinium-based contrast agents—what is the evidence for ‘gadolinium deposition disease’ and the use of chelation therapy? Clin Toxicol 58:151–160. 10.1080/15563650.2019.1681442 10.1080/15563650.2019.1681442 31663374

[17] Pasquini L, Napolitano A, Pignatelli M et al (2022) Synthetic post-contrast imaging through artificial intelligence: clinical applications of virtual and augmented contrast media. Pharmaceutics 14:2378. 10.3390/pharmaceutics14112378 36365197 10.3390/pharmaceutics14112378 PMC9695136

[18] Fringuello Mingo A, Colombo Serra S, Macula A et al (2023) Amplifying the effects of contrast agents on magnetic resonance images using a deep learning method trained on synthetic data. Invest Radiol 58:853–864. 10.1097/RLI.0000000000000998 37378418 10.1097/RLI.0000000000000998 PMC10662587

[19] Caravan P (2024) Divalent manganese complexes as potential replacements for gadolinium-based contrast agents. Invest Radiol 59:187–196. 10.1097/RLI.0000000000001053 38038701 10.1097/RLI.0000000000001053 PMC10841418

[20] Nyström NN, Liu H, Martinez FM et al (2022) Gadolinium-free magnetic resonance imaging of the liver *via* an Oatp1-targeted manganese(III) porphyrin. J Med Chem 65:9846–9857. 10.1021/acs.jmedchem.2c00500 10.1021/acs.jmedchem.2c00500 35852350

[21] Jeon M, Halbert MV, Stephen ZR et al (2021) Iron oxide nanoparticles as *T*_1_ contrast agents for magnetic resonance imaging: fundamentals, challenges, applications, and prospectives. Adv Mater 33:1906539. 10.1002/adma.201906539 10.1002/adma.201906539 PMC8022883 32495404

[22] Marasini R, Rayamajhi S, Moreno-Sanchez A et al (2021) Iron(iii) chelated paramagnetic polymeric nanoparticle formulation as a next-generation *T*_1_-weighted MRI contrast agent. RSC Adv 11:32216–32226. 10.1039/D1RA05544E 35495502 10.1039/d1ra05544e PMC9041822

[23] Boehm-Sturm P, Haeckel A, Hauptmann R et al (2018) Low-molecular-weight iron chelates may be an alternative to gadolinium-based contrast agents for T1-weighted contrast-enhanced MR imaging. Radiology 286:537–546. 10.1148/radiol.2017170116 28880786 10.1148/radiol.2017170116

[24] Ward KM, Aletras AH, Balaban RS (2000) A new class of contrast agents for MRI based on proton chemical exchange dependent saturation transfer (CEST). J Magn Reson 143:79–87. 10.1006/jmre.1999.1956 10698648 10.1006/jmre.1999.1956

[25] Wei Y, Zhao M, Yang F et al (2016) Iron overload by superparamagnetic iron oxide nanoparticles is a high risk factor in cirrhosis by a systems toxicology assessment. Sci Rep 6:29110. 10.1038/srep29110 27357559 10.1038/srep29110 PMC4928111

[26] Wang Y-XJ (2015) Current status of superparamagnetic iron oxide contrast agents for liver magnetic resonance imaging. World J Gastroenterol 21:13400. 10.3748/wjg.v21.i47.13400 26715826 10.3748/wjg.v21.i47.13400 PMC4679775

[27] Ni F, Haeckel A, Hojjat H et al (2025) Comparison of iron(III)-trans-1,4-diaminocyclohexane-*t*CDTA and iron(III)-trans-1,4-diaminocyclohexane-*t*CDTA-dimer with gadobutrol for T1 contrast enhancement in DCE-MRI. Invest Radiol. 10.1097/RLI.0000000000001172 10.1097/RLI.0000000000001172 40048247

[28] Mi H, Boehm-Sturm P, Haeckel A et al (2025) High-resolution quantitative mapping of extracellular pH by ratiometric MRI with iron chelates in a tumor mouse model. Radiol Med. 10.1007/s11547-025-02020-z 10.1007/s11547-025-02020-z PMC12367910 40392403

[29] Xie J, Haeckel A, Hauptmann R et al (2021) Iron(III)- *t*CDTA derivatives as MRI contrast agents: increased T_1_ relaxivities at higher magnetic field strength and pH sensing. Magn Reson Med 85:3370–3382. 10.1002/mrm.28664 33538352 10.1002/mrm.28664

[30] Laurent S, Vander Elst L, Henoumont C et al (2010) How to measure the transmetallation of a gadolinium complex. Contrast Media Mol Imaging 5:305–308. 10.1002/cmmi.388 20803503 10.1002/cmmi.388

[31] Lessing PA, Erickson AW (2003) Synthesis and characterization of gadolinium phosphate neutron absorber. J Eur Ceram Soc 23:3049–3057. 10.1016/S0955-2219(03)00100-6

[32] Paton C, Hellstrom J, Paul B et al (2011) Iolite: freeware for the visualisation and processing of mass spectrometric data. J Anal Spectrom 26:2508. 10.1039/c1ja10172b

[33] Paul B, Petrus J, Savard D et al (2023) Time resolved trace element calibration strategies for LA-ICP-MS. J Anal Spectrom 38:1995–2006. 10.1039/D3JA00037K

[34] Schannor M, Oelze M, Traub H et al (2025) Advancing biomarker research: *in situ* Cu isotope analysis in liver tumors by LA-MC-ICP-MS. Anal Chem 97:4425–4432. 10.1021/acs.analchem.4c05626 39964051 10.1021/acs.analchem.4c05626 PMC11883731

[35] McRae SW, Cleary M, DeRoche D et al (2023) Development of a suite of gadolinium-free OATP1-targeted paramagnetic probes for liver MRI. J Med Chem 66:6567–6576. 10.1021/acs.jmedchem.2c01561 37159947 10.1021/acs.jmedchem.2c01561 PMC12074583

[36] Gaeta M, Galletta K, Cavallaro M et al (2024) T1 relaxation: chemo-physical fundamentals of magnetic resonance imaging and clinical applications. Insights Imaging 15:200. 10.1186/s13244-024-01744-2 39120775 10.1186/s13244-024-01744-2 PMC11315875

[37] De León-Rodríguez LM, Martins AF, Pinho MC et al (2015) Basic MR relaxation mechanisms and contrast agent design: MR relaxation mechanisms and contrast agents. J Magn Reson Imaging 42:545–565. 10.1002/jmri.24787 25975847 10.1002/jmri.24787 PMC4537356

[38] Asik D, Smolinski R, Abozeid SM et al (2020) Modulating the properties of Fe(III) macrocyclic MRI contrast agents by appending sulfonate or hydroxyl groups. Molecules 25:2291. 10.3390/molecules25102291 32414058 10.3390/molecules25102291 PMC7288058

[39] Tweedle MF (2021) Alternatives to gadolinium-based contrast agents. Invest Radiol 56:35–41. 10.1097/RLI.0000000000000725 32932378 10.1097/RLI.0000000000000725

[40] Emsley J (2011) Nature’s building blocks: an A-Z guide to the elements. Oxford University Press, New York

[41] Frydrychowicz A, Lubner MG, Brown JJ et al (2012) Hepatobiliary MR imaging with gadolinium-based contrast agents. Magn Reson Imaging 35:492–511. 10.1002/jmri.22833 10.1002/jmri.22833 PMC3281562 22334493

[42] Jia J, Puls D, Oswald S et al (2014) Characterization of the intestinal and hepatic uptake/efflux transport of the magnetic resonance imaging contrast agent gadolinium-ethoxylbenzyl-diethylenetriamine-pentaacetic acid. Invest Radiol 49:78–86. 10.1097/RLI.0b013e3182a70043 24056116 10.1097/RLI.0b013e3182a70043

[43] McFeely SJ, Ritchie TK, Yu J et al (2019) Identification and evaluation of clinical substrates of organic anion transporting polypeptides 1B1 and 1B3. Clin Transl Sci 12:379–387. 10.1111/cts.12623 30706983 10.1111/cts.12623 PMC6662428

[44] Kullak-Ublick GA, Ismair MG, Stieger B et al (2001) Organic anion-transporting polypeptide B (OATP-B) and its functional comparison with three other OATPs of human liver. Gastroenterology 120:525–533. 10.1053/gast.2001.21176 11159893 10.1053/gast.2001.21176

[45] Hagenbuch B, Stieger B, Locher KP (2025) Organic anion transporting polypeptides: pharmacology, toxicology, structure, and transport mechanisms. Pharmacol Rev 77:100023. 10.1016/j.pharmr.2024.100023 40148036 10.1016/j.pharmr.2024.100023 PMC13095437

[46] Zelcer N, Saeki T, Reid G et al (2001) Characterization of drug transport by the human multidrug resistance protein 3 (ABCC3). J Biol Chem 276:46400–46407. 10.1074/jbc.M107041200 11581266 10.1074/jbc.M107041200

[47] Kinzi J, Grube M, Meyer Zu Schwabedissen HE (2021) OATP2B1—the underrated member of the organic anion transporting polypeptide family of drug transporters? Biochem Pharmacol 188:114534. 10.1016/j.bcp.2021.114534 33794186 10.1016/j.bcp.2021.114534

[48] Toth GB, Varallyay CG, Horvath A et al (2017) Current and potential imaging applications of ferumoxytol for magnetic resonance imaging. Kidney Int 92:47–66. 10.1016/j.kint.2016.12.037 28434822 10.1016/j.kint.2016.12.037 PMC5505659

[49] Nguyen K-L, Yoshida T, Kathuria-Prakash N et al (2019) Multicenter safety and practice for off-label diagnostic use of ferumoxytol in MRI. Radiology 293:554–564. 10.1148/radiol.2019190477 31638489 10.1148/radiol.2019190477 PMC6884068

[50] He L, Wang H, Zeng Z et al (2024) Rigid Fe(III) chelate with phosphonate pendants: a stable and effective extracellular MRI contrast agent. J Med Chem 67:8630–8641. 10.1021/acs.jmedchem.3c02338 38747630 10.1021/acs.jmedchem.3c02338

[51] Tamada T, Ito K, Sone T et al (2011) Gd-EOB-DTPA enhanced MR imaging: evaluation of biliary and renal excretion in normal and cirrhotic livers. Eur J Radiol 80:e207–e211. 10.1016/j.ejrad.2010.08.033 20869827 10.1016/j.ejrad.2010.08.033

[52] Kirchin MA, Lorusso V, Pirovano G (2015) Compensatory biliary and urinary excretion of gadobenate ion after administration of gadobenate dimeglumine (MultiHance®) in cases of impaired hepatic or renal function: a mechanism that may aid in the prevention of nephrogenic systemic fibrosis? Br J Radiol 88:20140526. 10.1259/bjr.20140526 25651409 10.1259/bjr.20140526 PMC4651256

