# Supplementary material for: [Fe-EOB-tCDTA] generates strong contrast in the blood but not in the liver, despite inhibiting cellular [Gd-EOB-DTPA]2- uptake and partial liver excretion
Source: Eur Radiol Exp. 2026 May 13;10:65. doi: 10.1186/s41747-026-00731-0 (PMC13172164; doi:10.1186/s41747-026-00731-0)
Supplement: Supplementary file 1 — Additional file 1: Fig. S1 HPLC results for EOB-tCDTA (a) and Fe-EOB-tCDTA before (b) and after (c) autoclaving. Method: gradient reverse phase; mobile phase: 2-66% acetonitrile with ammonium bicarbonate at PH 7.8. Flow rate: 1.0 mL/min for 20 min. The retention time of EOB-tCDTA was 8.49 min and the main peak area was 98.26%. The retention times of Fe-EOB-tCDTA before and after sterilization were 2.13 and 2.26 min, respectively. Fig. S2 MALDI Mass Spectrometry for EOB-tCDTA Expected mass: 479.56 g/mol. Theoretical masses: [EOB-tCDTA+H]+: 480.56 g/mol; [EOB-tCDTA+Na]+: 502.56 g/mol. Fig. S3 Microscopic images taken before LA-ICP-MSI of Fe- EOB-tCDTA and Gd-EOB-DTPA in BRL-3A cells. BRL-3A cells were treated with Gd-EOB-DTPA only or with Gd-EOB-DTPA and Fe-EOB-tCDTA at different ratios (1:0.5, 1:1, 1:2.5, 1:5, 1:10, and 1:20). Fig. S4 Spectral light absorption curves of Fe-EOB-tCDTA (a) and [Fe-(tCDTA)]-(b) during zinc challenge over 9 h. Absorption measurements were performed immediately after mixing with the [ZnCl2 Na2HPO4] solution. The same [ZnCl2 Na2HPO4] solution was used as a reference for all measurements. The wavelength of 410 nm was chosen for the time curves in Figure 3. Fig. S5 T1 contrast enhancement of Fe-EOB-tCDTA and Gd-EOB-DTPA in mice compared with that of Gd-DO3A-butrol. (a) Relative signal enhancement time curves of the cardiac left ventricle, liver, kidney, and muscle after intravenous injection of contrast agents. (b) Comparison of peak relative enhancement in the cardiac left ventricle, liver, kidney, and muscle of mice after contrast agent injection, with significance indicated. Statistical analysis was performed using one-way ANOVA with Dunnett's multiple comparisons (Gd-DO3A-butrol as control). ANOVA, analysis of variance. Table S1 DCE-MRI properties of Fe-EOB-tCDTA and Gd-EOB-DTPA in the gallbladder, muscle, and cerebrum. [file 41747_2026_731_MOESM1_ESM.pdf]

[Fe-EOB-tCDTA] generates strong contrast in the blood but not in the liver, despite inhibiting cellular [Gd-EOB-DTPA]2- uptake and partial liver excretion

ELECTRONIC SUPPLEMENTARY MATERIAL

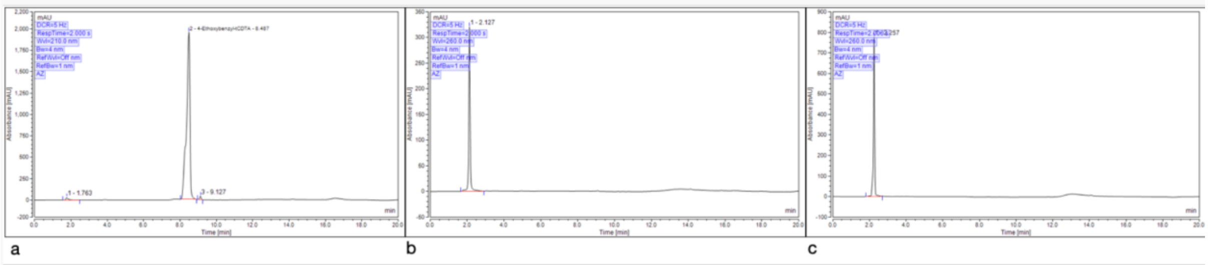

Supplemental Fig. S1: HPLC results for EOB-*t*CDTA (a) and Fe-EOB-*t*CDTA before (b) and after (c) autoclaving. Method: gradient reverse phase; mobile phase: 2-66% acetonitrile with ammonium bicarbonate at PH 7.8. Flow rate: 1.0 mL/min for 20 min. The retention time of EOB-*t*CDTA was 8.49 min and the main peak area was 98.26%. The retention times of Fe-EOB-*t*CDTA before and after sterilization were 2.13 and 2.26 min, respectively.

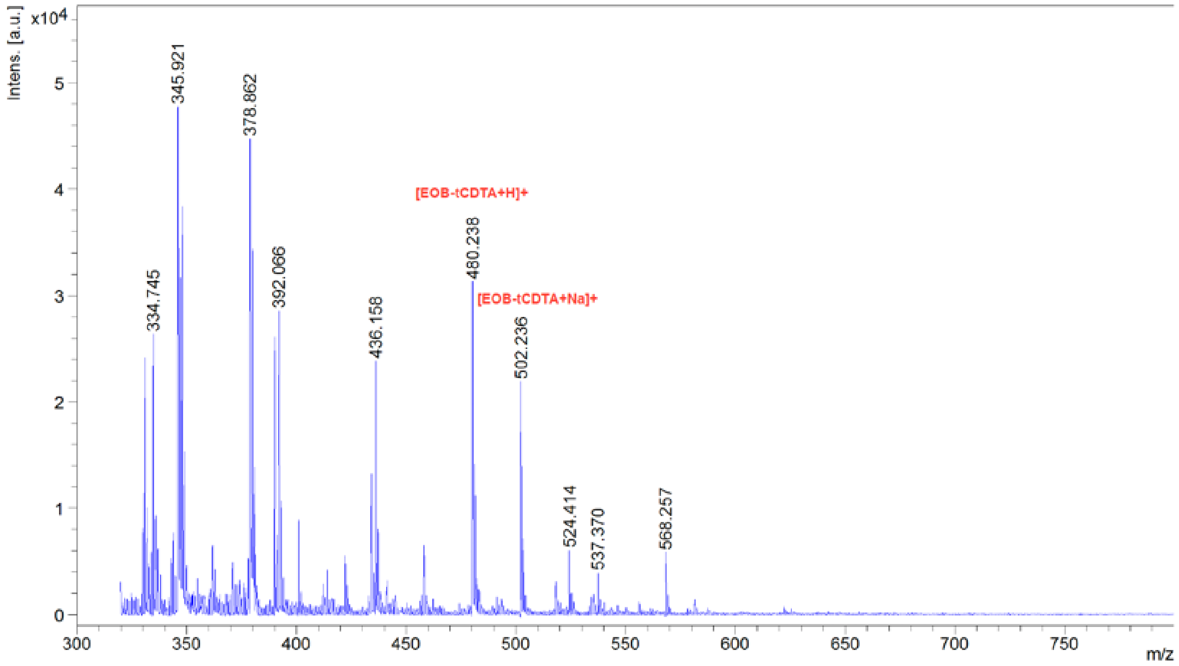

Supplemental Fig. S2: MALDI Mass Spectrometry for EOB-*t*CDTA Expected mass: 479.56 g/mol. Theoretical masses: [EOB-*t*CDTA+H]$^+$: 480.56 g/mol; [EOB-*t*CDTA+Na]$^+$: 502.56 g/mol.

Eur Radiol Exp (2026) Ni F, Haeckel A, Hojjat H, Schannor M, Traub H, Schellenberger E.

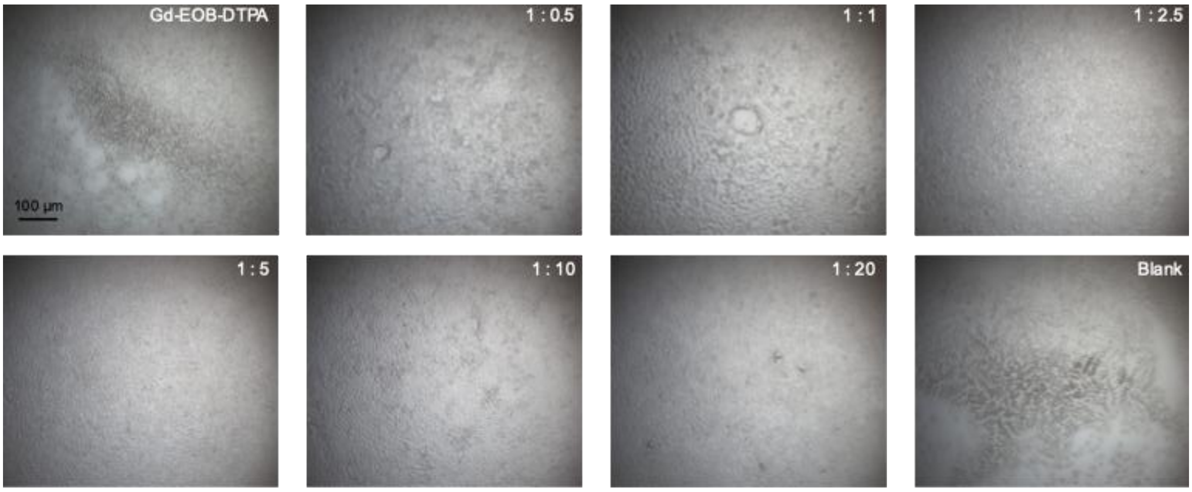

Supplemental Fig. S3: Microscopic images taken before LA-ICP-MSI of Fe-EOB-*t*CDTA and Gd-EOB-DTPA in BRL-3A cells. BRL-3A cells were treated with Gd-EOB-DTPA only or with Gd-EOB-DTPA and Fe-EOB-*t*CDTA at different ratios (1:0.5, 1:1, 1:2.5, 1:5, 1:10, and 1:20).

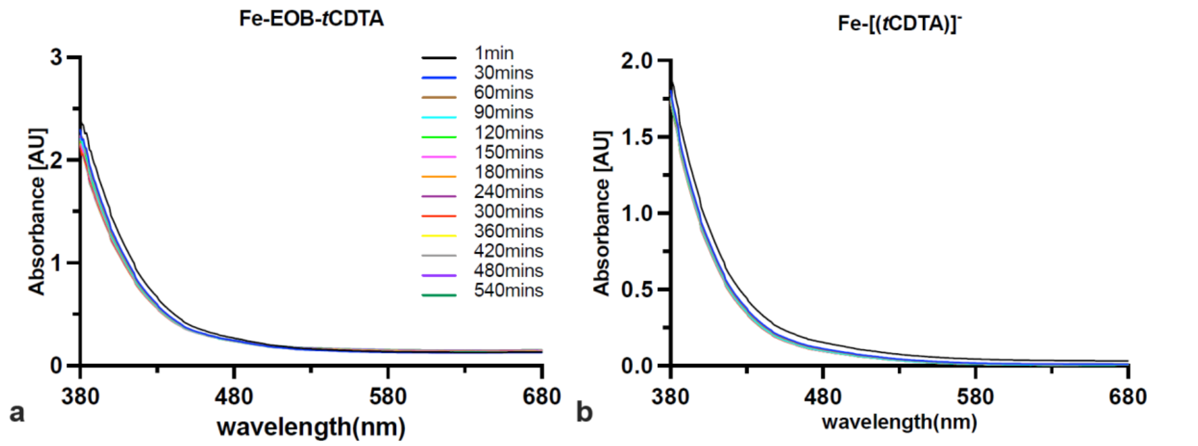

Supplemental Fig. S4: Spectral light absorption curves of Fe-EOB-*t*CDTA (a) and [Fe-(*t*CDTA)]$^-$(b) during zinc challenge over 9 h. Absorption measurements were performed immediately after mixing with the [$ZnCl_2$ Na_2HPO_4] solution. The same [$ZnCl_2$ Na_2HPO_4] solution was used as a reference for all measurements. The wavelength of 410 nm was chosen for the time curves in Figure 3.

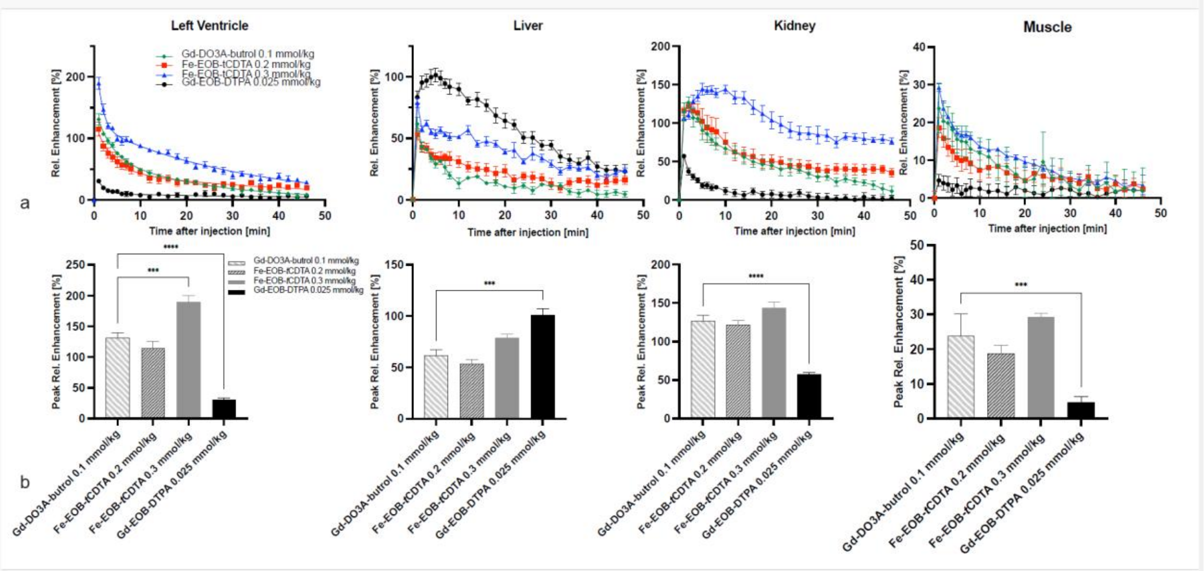

Supplemental Fig. S5: T1 contrast enhancement of Fe-EOB-*t*CDTA and Gd-EOB-DTPA in mice compared with that of Gd-DO3A-butrol. (a) Relative signal enhancement time curves of the cardiac left ventricle, liver, kidney, and muscle after intravenous injection of contrast agents. (b) Comparison of peak relative enhancement in the cardiac left ventricle, liver, kidney, and muscle of mice after contrast agent injection, with significance indicated. Statistical analysis was performed using one-way ANOVA with Dunnett's multiple comparisons (Gd-DO3A-butrol as control). ANOVA, analysis of variance.

Supplemental Table 1 DCE-MRI properties of Fe-EOB-*t*CDTA and Gd-EOB-DTPA in the gallbladder, muscle, and cerebrum.

Parameter	Gd-EOB-DTPA 0.025 mmol/kg	Fe-EOB-*t*CDTA 0.2 mmol/kg	Fe-EOB-*t*CDTA 0.3 mmol/kg
SI predose [SI_{pre}]	156.1 ± 3.13/283.3 ± 5.01/318.0 ± 2.87	180.9 ± 6.99/294.7 ± 5.01/324.7 ± 7.19	183.0 ± 3.63/292.2 ± 6.55/329.2 ± 7.93
SI postdose [SI_{post}]	678.9 ± 44.85/296.6 ± 4.52/342.3 ± 8.64	713.5 ± 33.38/349.8 ± 7.53/345.6 ± 7.70	807.6 ± 23.71/377.8 ± 3.12/360.3 ± 5.65
Peak RE [%]	334.8 ± 28.73/6.63 ± 0.52/5.4 ± 1.22	294.3 ± 18.45/18.66 ± 0.88/6.92 ± 0.51	341.2 ± 12.95/29.57 ± 2.89/10.28 ± 2.05
AUC_{0-46} [%·min]	-/89.97 ± 34.21/167.7 ± 16.38	-/293.3 ± 36.96/235.2 ± 35.82	-/445.5 ± 18.53/359.8 ± 30.08
Washout Slope [%/min]	-/-0.04 ± 0.02/-0.01 ± 0.01	-/-0.26 ± 0.03/-0.03 ± 0.014	-/-0.45 ± 0.14/-0.06 ± 0.02

SI postdose: peak signal intensity after injection; AUC_{0-46}, area under the curve, 0-46 min; RE, relative enhancement. Data shown as gallbladder/muscle/cerebrum, mean ± SE.
